# Re-description of seven predatory mite species of family Phytoseiidae (Acari: Mesostigmata) sourced from Florida citrus groves

**DOI:** 10.1371/journal.pone.0255455

**Published:** 2021-08-20

**Authors:** Emilie P. Demard, Ismail Döker, Jawwad A. Qureshi

**Affiliations:** 1 Department of Entomology and Nematology, IFAS, Indian River Research and Education Center, University of Florida, Fort Pierce, FL, United States of America; 2 Agricultural Faculty, Plant Protection Department, Acarology Laboratory, Çukurova University, Adana, Turkey; 3 Department of Entomology and Nematology, IFAS, Southwest Florida Research and Education Center, University of Florida, Immokalee, FL, United States of America; Laboratoire de Biologie du Développement de Villefranche-sur-Mer, FRANCE

## Abstract

Predatory mites in the family Phytoseiidae (Acari: Mesostigmata) are of great importance as biological control agents of pest mites and other arthropods. Correct identification of species is crucial to implement effective biological control of target pests. Here, we provide re-descriptions of seven phytoseiid mite species collected from citrus orchards in Florida. The several important morphological features including dorsal setae lengths, dorsal solenostomes, shape of calyx of spermatheca, chelicera dentition, measurements, and shape of macrosetae on legs currently used to discriminate phytoseiid species were missing in the original descriptions and re-descriptions of these species. Additionally, we observed the presence of a previously unnoted taxonomically important character on *Proprioseius meridionalis* Chant. Therefore, the re-description was essential for further diagnosis of this species. Accordingly, the validity of the presence/absence of this structure as a diagnostic character to separate species groups in the genus *Proprioseius* should be re-considered. Furthermore, *Typhlodromalus peregrinus*, a species for which a series of morphological variations are reported in previous descriptions, is re-described and illustrated from Clermont, Florida, a location very close (10 km) to its type location (Minneola), and the leaves of type host citrus. The macrosetae StIV was knobbed apically in all our specimens of *T*. *peregrinus* indicating invalidity of sharp-pointed or knobbed StIV to separate this species from a closely related species, *T*. *aripo* De Leon. These re-descriptions and species are important to utilizing authentic and promising candidates for biological control.

## Introduction

Predatory mites in the family Phytoseiidae (Acari: Mesostigmata) are important biological control agents of many pest species that include spider mites, thrips, and whiteflies [1, 2]. They may also attack the relatively recently introduced Asian citrus psyllid, *Diaphorina citri* Kuwayama (Hemiptera: Liviidae), responsible for vectoring the causal pathogens of huanglongbing or citrus greening disease that is currently spreading across Florida and other states [3, 4]. The phytoseiid species such as *Amblydromalus limonicus* (Garman & McGregor), *Amblyseius andersoni* (Chant), *A*. *swirskii* Athias-Henriot, *Neoseiulus barkeri* Hughes, *N*. *californicus* (McGregor), *N*. *cucumeris* (Oudemans), *N*. *fallacis* (Garman), *Transeius montdorensis* (Schicha) and *Phytoseiulus persimilis* Athias-Henriot are currently reared on a commercial scale and utilized to control several pests all over the world [5]. Determination of the identity of native populations of predatory mites is of considerable importance for achieving effective local pest control. Indigenous populations or species can often provide greater control success in the specific region and environmental conditions to which they are adapted [6].

Phytoseiid mite fauna in the United States has been well documented with about 370 species [7–11]. A total of 140 species, including synonyms, have been reported from Florida [1, 11, 12]. Studies on phytoseiid taxonomy from Florida date back to the mid-1950s with a series of new species being described between 1955 to 2011 [1, 13–17]. However, most of these descriptions or re-descriptions did not include many crucial taxonomical characters which are currently used to discriminate phytoseiid species such as dorsal setae lengths, dorsal solenostomes, shape of calyx of spermatheca, chelicera dentition, and measurements and shape of macrosetae on legs. The inadequate descriptions and re-descriptions or the absence of re-descriptions for some phytoseiid species have made accurate species identifications a challenge. This situation has often resulted in misidentifications and overall confusion among taxonomists.

We have been collecting predatory mites from multiple citrus groves in Florida, to find and identify species for biological control in citrus crops. The older descriptions or re-descriptions of some species lacked some important morphological features for species identification. Those did not include the necessary details for species separation, and therefore do not meet the current standards. We provide the re-description of seven phytoseiid mite species collected from multiple locations in Florida and important for biological control.

## Materials and methods

### Mite species and locations of collection

Permission was sought and granted from the orchard owners prior to undertaking all field collecting for this study. Information on the species, their habitat, and the location of the collection are provided in Table 1.

**Table 1:** Species, number of females, habitat, location, coordinates, altitude, and date of collection of the redescribed specimens.

Species	Females (n)	Habitat	Location	Latitude Longitude	Altitude	Date of collection
*Neoseiulus marinellus* (Muma)	2	Leaf litter	Clermont	28°36’35.9"N 81°44’59.8"W	45 m	December 11, 2019
	2	Ground cover	Vero Beach	27°39’17.0"N 80°27’49.6"W	7 m	July 31, 2019
	1	Leaf litter	Vero Beach	27°35’23.8"N 80°37’11.5"W	9 m	February 4, 2020
*Neoseiulus planatus* (Muma)	1	Ground cover	Vero Beach	27°39’17.0"N 80°27’49.6"W	7 m	July 31, 2019
*Proprioseius meridionalis* Chant	5	Ground cover	Clermont	28°36’35.9"N 81°44’59.8"W	45 m	September 30, 2019
*Amblyseius aerialis* (Muma)	1	Ground cover	Clermont	28°36’35.9"N 81°44’59.8"W	45 m	August 5, 2019
	2	Citrus leaves	Fort Pierce	27°26’08.3"N 80°26’49.1"W	8 m	June 6, 2019
	1	Leaf litter	Fort Pierce	27°23’27.7"N 80°27’50.5"W	7 m	January 29, 2020
	1	Citrus leaves	Fort Pierce	27°26’08.3"N 80°26’49.1"W	8 m	February 13, 2020
	1	Leaf litter	Vero Beach	27°35’23.8"N 80°37’11.5"W	9 m	February 4, 2020
*Amblyseius curiosus* (Chant & Baker)	1	Leaf litter	Clermont	28°36’35.9"N 81°44’59.8"W	45 m	February 3, 2020
	3	Leaf litter	Vero Beach	27°39’17.0"N 80°27’49.6"W	7 m	June 10, 2019
	1	Leaf litter	Vero Beach	27°39’17.0"N 80°27’49.6"W	7 m	January 29, 2020
*Proprioseiopsis carolinianus* (Muma, Metz & Farrier)	3	Leaf litter	Fort Pierce	27°30’28.3"N 80°36’50.4"W	8 m	July 30, 2019
	2	Leaf litter	Fort Pierce	27°30’28.3"N 80°36’50.4"W	8 m	November 26, 2019
	2	Leaf litter	Fort Pierce	27°23’27.7"N 80°27’50.5"W	7 m	January 29, 2020
	2	Leaf litter	Fort Pierce	27°30’28.3"N 80°36’50.4"W	8 m	January 30, 2020
	2	Leaf litter	Vero Beach	27°39’17.0"N 80°27’49.6"W	7 m	June 10, 2019
	1	Leaf litter	Vero Beach	27°35’23.8"N 80°37’11.5"W	9 m	December 12, 2019
	1	Leaf litter	Vero Beach	27°35’23.8"N 80°37’11.5"W	9 m	February 4, 2020
	1	Leaf litter	Vero Beach	27°39’17.0"N 80°27’49.6"W	7 m	February 29, 2020
*Typhlodromalus peregrinus* (Muma)	2	Ground cover	Clermont	28°36’35.9"N 81°44’59.8"W	45 m	June 3, 2019
	1	Ground cover	Clermont	28°36’35.9"N 81°44’59.8"W	45 m	September 30, 2019
	2	Ground cover	Clermont	28°36’35.9"N 81°44’59.8"W	45 m	February 3, 2020
	1	Ground cover	Clermont	28°39’36.6"N 81°44’52.3"W	63 m	February 3, 2020

### Extraction of mites from samples

The ground cover and canopy leaf samples were washed in a jar filled with 250 ml of 80% ethanol. The jar was shaken for about 30 seconds to dislodge the mites. The vegetative material from the jars was then retrieved using forcep and discarded. Leaf litter samples were collected in a four-gallon plastic bag and then processed through the Berlese funnel (Collapsible Berlese funnel, BioQuip, CA, USA) and stored in 80% ethanol. The mites were kept in 60% lactic acid for 24 hours at 55°C.

### Mounting and examination of the specimens

Specimens were mounted on microscope slides using Hoyer’s medium and dried on a slide warmer at 50°C (Premiere® XH-2004) for five days. Further examinations were conducted using an Olympus® CX-41 microscope. Pictures of type specimens were taken with a Leica® DMC2900 camera mounted on a Leica® DM1000 LED microscope and a Zeiss AxioCam ICc5 camera mounted on a Zeiss Axio Imager D1 microscope. The taxonomic system is based on that proposed by Chant & McMurtry [18]. The dorsal and ventral setal pattern notations follow Chant & Yoshida-Shaul [19–21]. Other terminologies follow Athias-Henriot [22, 23] for organotaxy, and Evans [24], and Evans & Till [25] for the ventral pores and leg chaetotaxy. The species redescribed were assigned consecutive numbers and arranged according to the commonly used taxonomic system worldwide [18]. After a species’ scientific name, the numbers in the parenthesis refer to the article and follow with the page number containing previously reported information on a particular species. Illustrations were prepared using an Olympus U-Da drawing attachment camera Lucida. Measurements are given in micrometers as mean followed by the range in parenthesis. Examined specimens were deposited into the mite collection of the Acarology Laboratory, Department of Plant Protection, Çukurova University, Adana, Turkey, and the Indian River Research Center, University of Florida, Fort Pierce, Florida, USA.

## Results and discussion

### Family Phytoseiidae Berlese, subfamily Amblyseiinae Muma Tribe Neoseiulini Chant & McMurtry, genus *Neoseiulus* Hughes

#### 1. *Neoseiulus marinellus* (Muma)

*Cydnodromus marinellus* [14]: 8.

*Neoseiulus marinellus* (Muma) [26]: 101; [27]: 667; [28]: 35; [1]: 147.

Female (n = 5).

**Fig 1:**
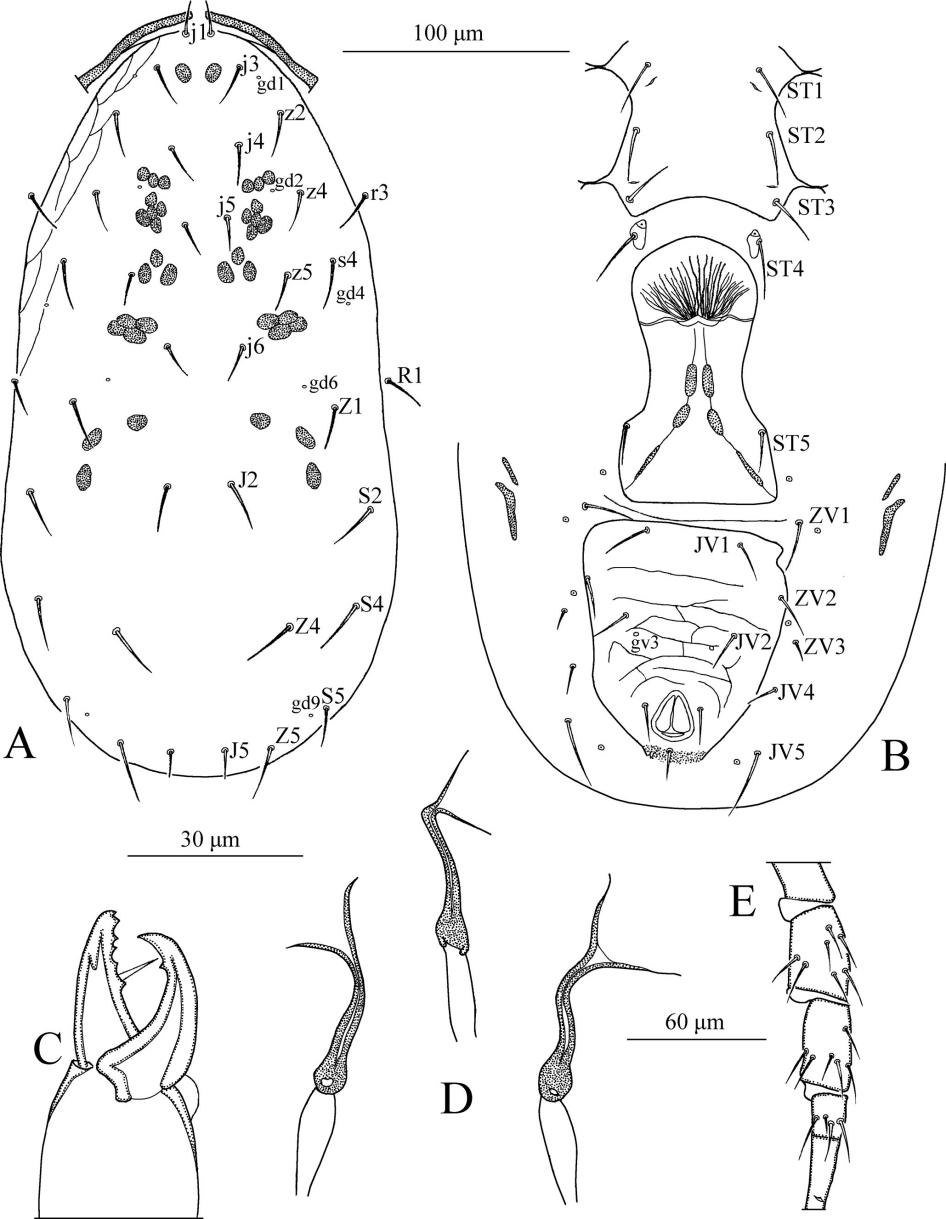
*Neoseiulus marinellus* (Muma)–Female (A–E): A. Dorsal shield, B. Ventral idiosoma, C. Chelicera, D. Spermatheca, E. Leg IV. Scale bars = 100 μm for A, B; 30 μm for C, D; 60 μm for E (Photo credit: Ismail Döker).

*Dorsum* (Fig 1A). Dorsal setal pattern 10A:9B (*r3* and *R1* off shield). Dorsal shield oval with a slight waist at the level of *Z1*, smooth except for a few anterolateral striations. Bearing five pairs of rounded solenostomes (*gd1*, *gd2*, *gd4*, *gd6*, and *gd9*). Muscle-marks (sigilla) visible on podosoma, length of dorsal shield 313 (293–325), width (distance between bases of *s4*) 143 (133–148), width (distance between bases of *S2*) 151 (141–155). All dorsal setae smooth. Measurements of dorsal setae as follows: *j1* 13 (10–14), *j3* 16 (12–18), *j4* 15 (14–16), *j5* 15 (15–16), *j6* 16 (15–17), *J2* 18 (17–20), *J5* 11 (11–12), *z2* 15 (14–16), *z4* 17 (16–17), *z5* 15 (14–16), *Z1* 16 (15–18), *Z4* 23 (22–24), *Z5* 24 (21–26), *s4* 16 (14–17), *S2* 19 (18–20), *S4* 18 (17–19), *S5* 16 (15–17), *r3* 15 (14–16), and *R1* 13 (11–15).

*Peritreme*. Long, extends beyond setae *j1*.

*Venter* (Fig 1B). Ventral setal pattern 14:JV–3:ZV. Sternal shield smooth, lightly sclerotized with three pairs of setae (*ST1*, *ST2*, *ST3*), two pairs of poroids (*pst1* and *pst2*). Distance (*ST1*–*ST3*) 59 (58–61), width (*ST2*–*ST2*) 61 (60–63). Metasternal setae *ST4* and a pair of pores (*pst3*) on metasternal shields. Genital shield smooth; width at level of genital setae (*ST5*) 58 (55–62). Ventrianal shield reticulated, bearing three pairs of pre-anal setae (*JV1*, *JV2*, and *ZV2*), a pair of para-anal (*Pa*) and a post-anal seta (*Pst*), and with a pair of small rounded solenostomes (*gv3*) posteromedian to *JV2*; distance *gv3*–*gv3* 33 (31–34). Length of ventrianal shield 103 (99–108), width at level of *ZV2* 83 (78–87), width at level of anus 68 (65–71). Setae *JV4*, *JV5*, *ZV1*, *ZV3*, and four pairs of poroids on integument surrounding ventrianal shield. Two pairs of metapodal plates, primary 20 (19–20), and secondary 11 (10–11) in length. Setae *JV5* smooth, 27 (26–28) in length.

*Chelicera* (Fig 1C). Fixed digit 29 long with five teeth, with *pilus dentilis*; movable digit 30 long with one tooth.

*Spermatheca* (Fig 1D). Calyx trumpet-like, elongated, flaring distally, 33 (31–34) in length; atrium nodular, enlarged, broadly joined to the calyx; major duct broad.

*Legs* (Fig 1E). Length of legs (base of coxae to base of claws) as follows: leg I 285 (280–290), leg II 213 (210–215), leg III 200 (195–205), leg IV 270 (265–275). Genua II, III, and IV each with seven setae. No macrosetae present on legs.

**Fig 2:**
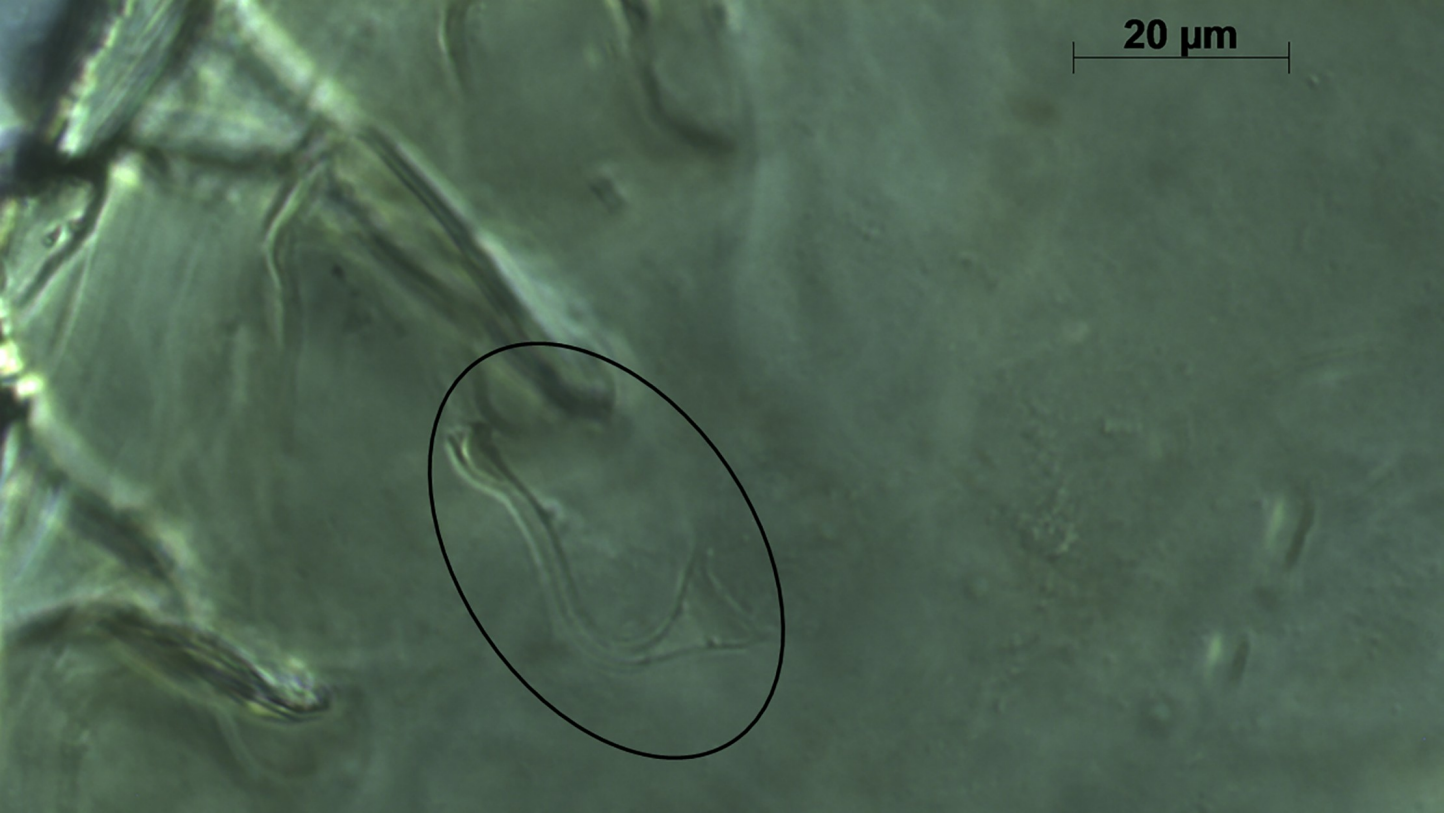
General view of spermatheca of *Neoseiulus marinellus* (holotype) (Photo credit: Ronald Ochoa).

*Remarks*. *Neoseiulus marinellus* description by Muma [14] was based on the material collected from leaf litter under *Citrus* sp. in Minneola, Florida. Many morphological details including measurements of dorsal setae were missing in the original description and subsequent re-descriptions [26, 27]. Here, we provide a complementary description of this species for the first time from specimens collected from citrus leaf litter from three locations in Florida. Morphological characters of the current specimens examined in this study are in agreement with those of type materials (Fig 2).

#### 2. *Neoseiulus planatus* (Muma)

*Cydnodromus planatus* [14]: 9.

*Neoseiulus planatus* (Muma); [26]: 104; [27]: 667; [28]: 37; [1]: 152.

Female (n = 1).

**Fig 3:**
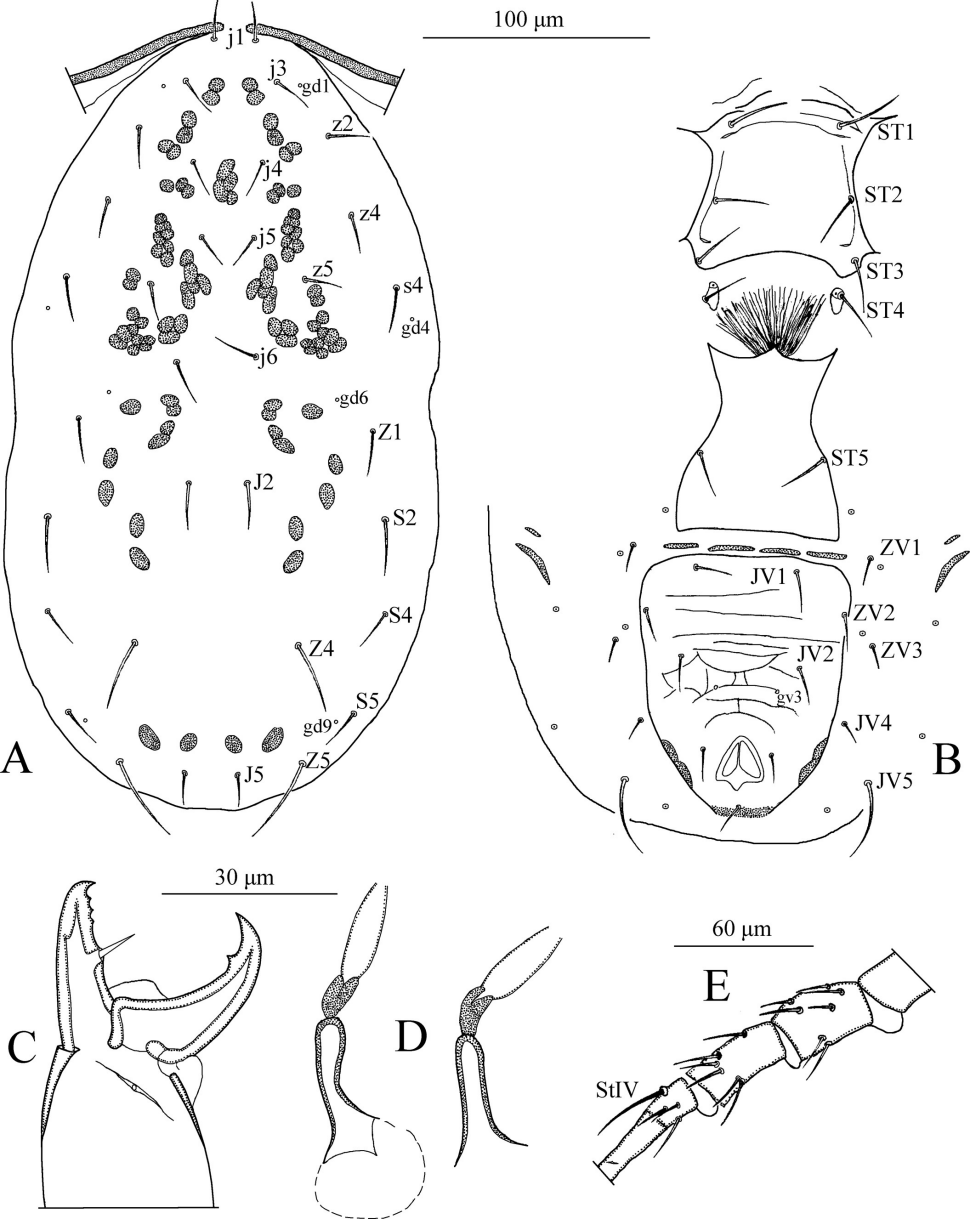
*Neoseiulus planatus* (Muma)–Female (A–E): A. Dorsal shield, B. Ventral idiosoma, C. Chelicera, D. Spermatheca, E. Leg IV. Scale bars = 100 μm for A, B; 30 μm for C, D; 60 μm for E (Photo credit: Ismail Döker).

*Dorsum* (Fig 3A). Dorsal setal pattern 10A:9B (*r3* and *R1* off shield). Dorsal shield oval with a slight waist at level of *Z1*, smooth. Bearing five pairs of rounded solenostomes (*gd1*, *gd2*, *gd4*, *gd6*, and *gd9*). Muscle-marks (sigilla) visible mostly on podosoma, length of dorsal shield 333, width (distance between bases of *s4*) 178, width (distance between bases of *S2*) 188. All dorsal setae smooth. Measurements of dorsal setae as follows: *j1* 15, *j3* 18, *j4* 17, *j5* 16, *j6* 17, *J2* 17, *J5* 14, *z2* 15, *z4* 18, *z5* 18, *Z1* 21, *Z4* 30, *Z5* 35, *s4* 23, *S2* 25, *S4* 21, *S5* 20, *r3* 18, and *R1* 17.

*Peritreme*. Long, extending beyond setae *j1*.

*Venter* (Fig 3B). Ventral setal pattern 14:JV–3:ZV. Sternal shield smooth except few anterior striations, sclerotized with three pairs of setae (*ST1*, *ST2*, *ST3*), two pairs of poroids (*pst1* and *pst2*). Distance (*ST1*–*ST3*) 60, width (*ST2*–*ST2*) 60. Metasternal setae *ST4* and a pair of pores (*pst3*) on metasternal shields. Genital shield smooth; width at level of genital setae (*ST5*) 58. Ventrianal shield reticulated, bearing three pairs of pre-anal setae (*JV1*, *JV2*, and *ZV2*), a pair of para-anal (*Pa*), and a post-anal seta (*Pst*), and with a pair of small rounded solenostomes (*gv3*) posteromedian to *JV2*; distance *gv3*–*gv3* 25. Length of ventrianal shield 115, width at level of *ZV2* 90, width at level of anus 75. Setae *JV4*, *JV5*, *ZV1*, *ZV3*, and six pairs of poroids on integument surrounding ventrianal shield. Setae *JV5* smooth, much longer than other ventral setae, 33 in length.

*Chelicera* (Fig 3C). Fixed digit 30 long with three apical teeth, with *pilus dentilis*; movable digit 30 long with one tooth.

*Spermatheca* (Fig 3D). Calyx saccular, elongated, flaring distally, 20 in length; atrium broad and forked at the juncture with major duct, slightly narrowing where it joins to the calyx; major duct very broad.

*Legs* (Fig 3E). Length of legs (base of coxae to base of claws) as follows: leg I 318, leg II 233, leg III 215, leg IV 313. Genua II, III, and IV each with seven setae. A short and sharp-pointed macrosetae is present on leg IV, *StIV* 33 in length.

**Fig 4:**
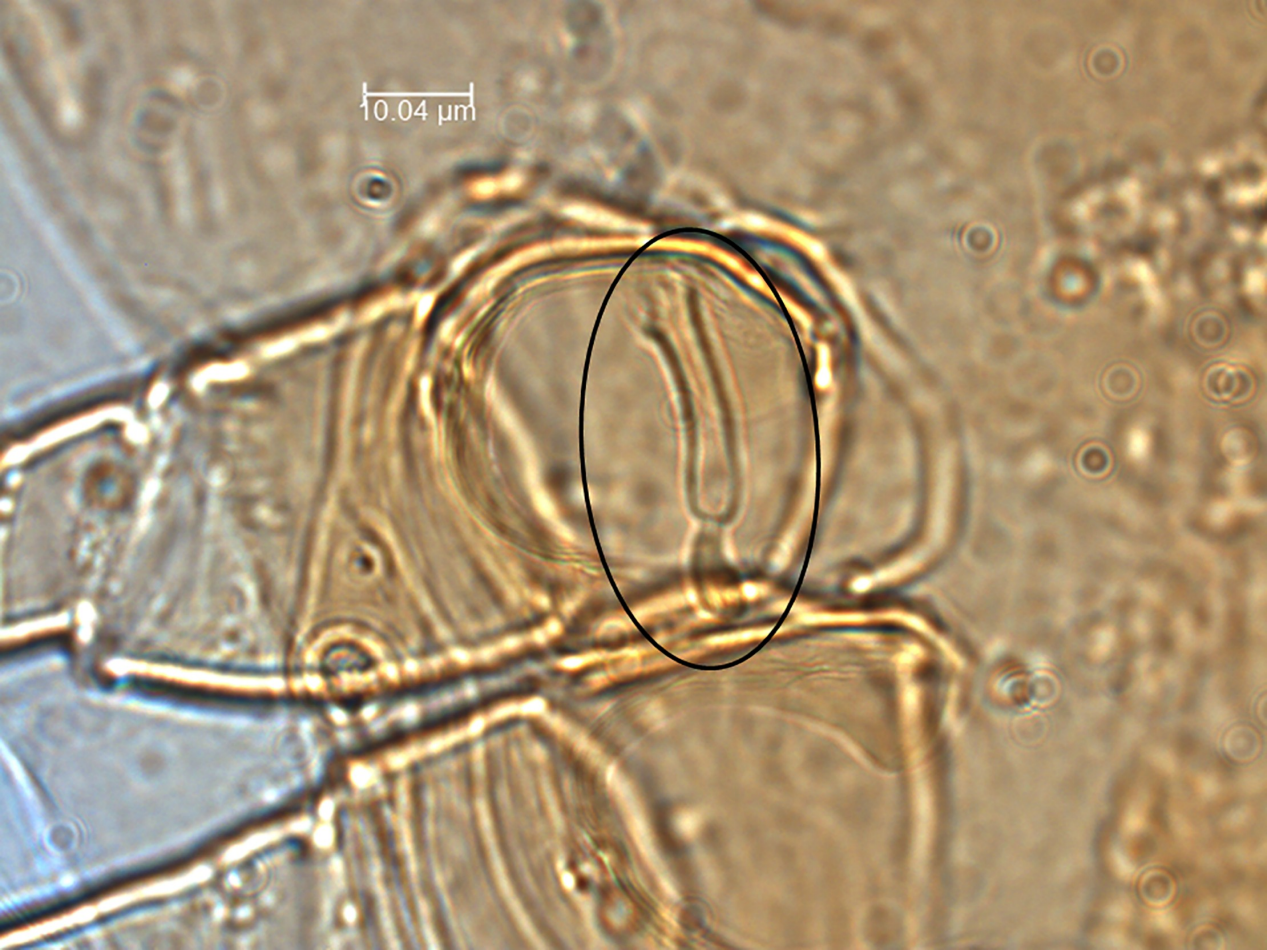
General view of spermatheca of *Neoseiulus planatus* from a specimen identified by Muma and collected in Florida (Photo credit: Emilie Demard).

*Remarks*. *Neoseiulus planatus* described by Muma [14], came from the material collected from leaf litter under *Citrus* sp. in Avon Park, Florida. Many morphological details of this species including measurements of dorsal setae were missing in the original description. The subsequent re-description by Muma & Denmark [26] contained relatively detailed drawings but was still missing the dorsal setae measurements. Most likely due to the inadequate descriptions, this species was treated as “uncertain species” in *barkeri* species group and *barkeri* species subgroup in the most recent revision of the genus by Chant & McMurtry [29]. Denmark & Evans [1] included several measurements such as *j3 (20)*, *z2 (20)*, *z4 (20)*, *Z4 (36)*, *Z5 (53)*, and calyx of spermatheca (21) in an identification key for the genus *Neoseiulus* found in North America and Hawaii. Their measurements match with those of the current specimen that we report except for *Z5* (35 *vs* 53). We consider this difference as intraspecific variation because the current specimen was collected approximately 100 km east of its type location. After examination of the spermatheca from a specimen identified by Muma in 1961, we found no differences with our specimen. Indeed, the calyx length between the specimens was the same (20 μm) (Fig 4). We also measured and found no difference in dorsal, ventral, and ventrianal setae between specimens. Unfortunately, the spermatheca was not obvious in the pictures sent by Dr. Ronald Ochoa to confirm it with the holotype.

### Tribe Kampimodromini Kolodochka, genus *Proprioseius* Chant

#### 3. *Proprioseius meridionalis* Chant

*Proprioseius meridionalis* [15]: 358; [30]: 111; [31]: 259; [26]: 25; [32]: 206; [1]: 229.

Female (n = 5).

**Fig 5:**
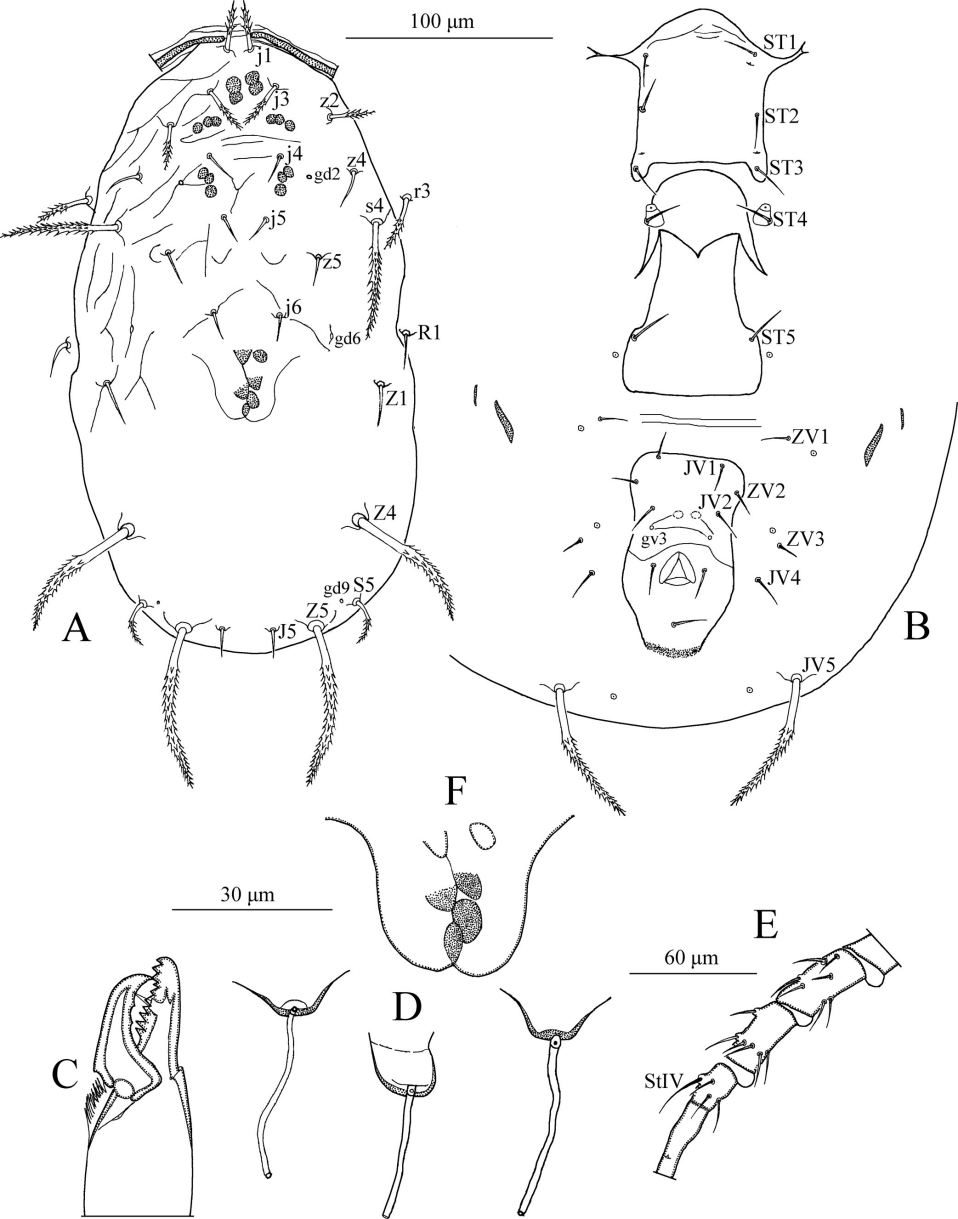
*Proprioseius meridionalis* Chant–Female (A–F): A. Dorsal shield, B. Ventral idiosoma, C. Chelicera, D. Spermatheca, E. Leg IV, F. Sub-cylindrical erect structure on dorsal shield. Scale bars = 100 μm for A, B; 30 μm for C, D, F; 60 μm for E (Photo credit: Ismail Döker).

*Dorsum* (Fig 5A, 5F). Dorsal setal pattern 10A:6E (*r3* and *R1* off shield). Dorsal shield oval with a slight waist at level of *R1*, strongly sculptured. Bearing three pairs of solenostomes (*gd2*, *gd6*, and *gd9*). Some muscle-marks (sigilla) visible on podosoma, a subcylindrical erect structure is located posterior to setae *j6*, length of dorsal shield 279 (273–285), width at level of *s4* 143 (138–150), width at level of *Z4* 147 (145–150). Dorsal setae *j1*, *j3*, *z2*, *Z4*, *Z5*, *s4*, *S5*, and *r3* are strongly serrated, and arises on tubercules. Other setae are simple, setae *z4*, *z5*, *Z1*, *J5* and *R1* also arise on tubercules. Measurements of dorsal setae as follows: *j1* 23 (20–25), *j3* 26 (25–28), *j4* 12 (10–13), *j5* 10, *j6* 13 (13–15), *J5* 12 (10–13), *z2* 23 (23–25), *z4* 18 (18–20), *z5* 10 (8–13), *Z1* 19 (18–20), *Z4* 63 (63–65), *Z5* 68 (65–70), *s4* 51 (50–53), *S5* 18 (15–20), *r3* 23(20–25), and *R1* 17 (15–18).

*Peritreme*. Long, extending to level setae *j1*.

*Venter* (Fig 5B). Ventral setal pattern 14:JV–3:ZV. Sternal shield smooth except few anterior striations, sclerotized with three pairs of setae (*ST1*, *ST2*, *ST3*), two pairs of poroids (*pst1* and *pst2*). Distance (*ST1*–*ST3*) 54 (53–55), width (*ST2*–*ST2*) 55 (53–58). Metasternal setae *ST4* and a pair of pores (*pst3*) on metasternal shields. Genital shield smooth; width at level of genital setae (*ST5*) 53 (50–55). Ventrianal shield smooth, elongated, much longer than wide, bearing three pairs of pre-anal setae (*JV1*, *JV2*, and *ZV2*), a pair of para-anal (*Pa*) and a post-anal seta (*Pst*), and with a pair of small rounded solenostomes (*gv3*) posteromedian to *JV2*; distance *gv3*–*gv3* 25. Length of ventrianal shield 95 (90–100), width at level of *ZV2* 49 (45–55), width at level of anus 48 (45–53). Setae *JV4*, *JV5*, *ZV1*, *ZV3*, and four pairs of poroids on integument surrounding ventrianal shield. Two pairs of metapodal plates, primary 23 (21–25), and secondary 11 (10–11) in length. Setae *JV5* serrated and arise on tubercules, much longer than other ventral setae, 53 (50–55) in length.

*Chelicera* (Fig 5C). Fixed digit 23 (23–25) long with nine strong teeth, with *pilus dentilis*; movable digit 26 (25–28) long with two teeth.

*Spermatheca* (Fig 5D). Calyx cup or dish-shaped, flaring distally at the base of vesicle, 8 (8–10) in length; atrium slightly nodular; major duct long.

*Legs* (Fig 5E). Length of legs (base of coxae to base of claws) as follows: leg I 265 (260–270), leg II 208 (200–215), leg III 202 (200–205), leg IV 292 (285–300). Genua II, III, and IV with eight, seven, and seven setae, respectively. A short and sharp-poined macrosetae that is noticeably thicker than other setae on the same segment, *StIV* 19 (18–20) in length.

**Fig 6:**
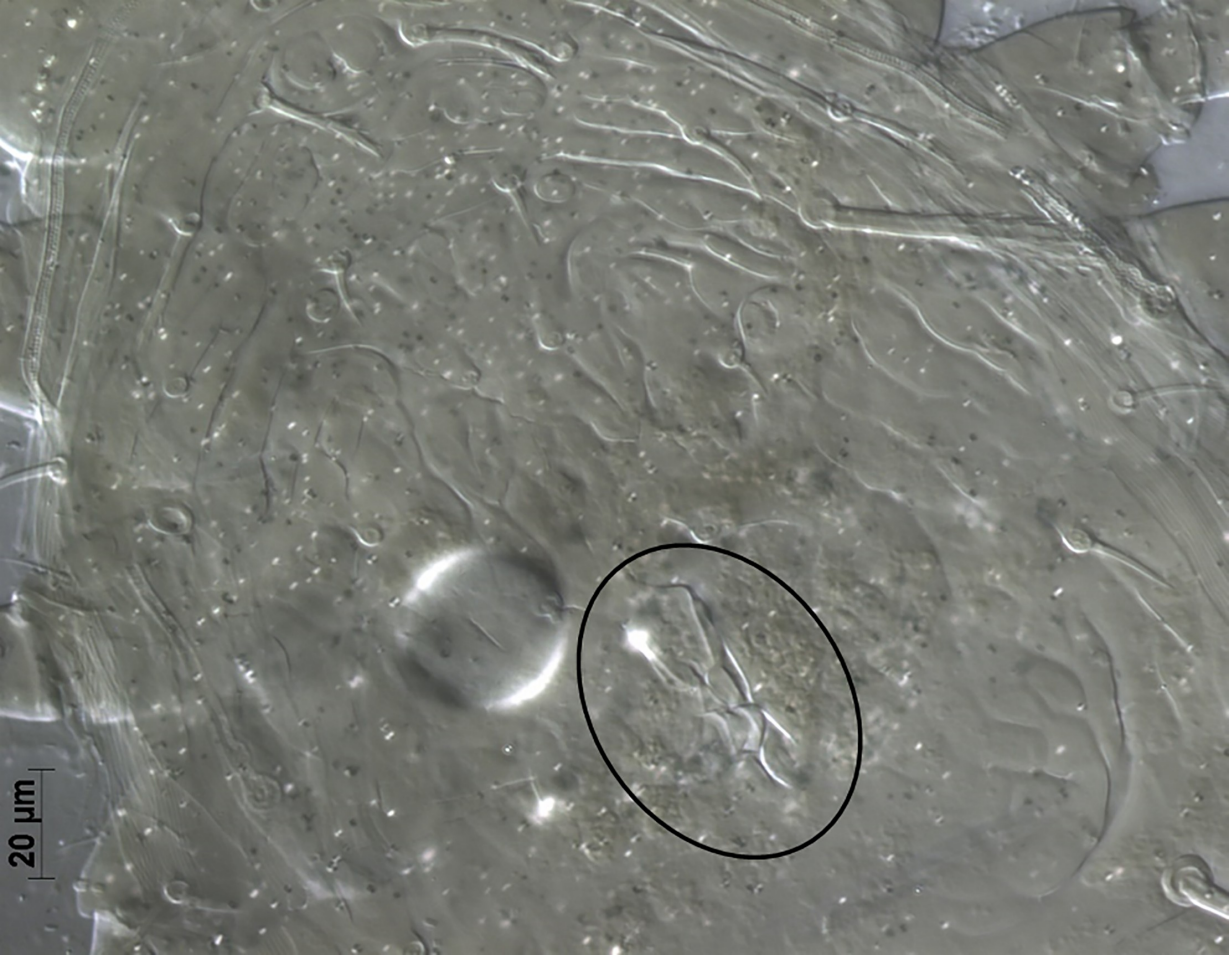
Subcylindrical erect structure on dorsal shield of *Proprioseius meridionalis* (holotype) (Photo credit: Ronald Ochoa).

*Remarks*. *Proprioseius meridionalis* described by Chant [15] came from the material collected from *Psychotria bahamensis* (Rubiaceae) in Homestead, Florida. In their revision of the tribe, Kampimodromini Kolodochka, Chant & McMurtry [32] divided genus *Proprioseius* into three species groups based on the relative length of lateral dorsal setae and presence/absence of subcylindrical, erect structure posterior to setae *j6*. They characterized *meridionalis* species group with the absence of this erect structure. However, we observed this erect structure in all specimens that we examined in our study. To ensure that the structure identified by Chant & McMurtry [32] was the one we observed in our samples, a specimen was also mounted laterally. As a result, we confirmed the presence of the erect structure. Finally, because all other morphological characters including setae measurements concurred with the original and the re-descriptions, we consider current specimens as *P*. *meridionalis* [15, 31]. We believe that this structure might have been overlooked in previous descriptions, probably due to ornamentations on the dorsal shield and poor optic material used at that time. Indeed, the presence of this structure in the type material was confirmed through communication with Dr. Ronald Ochoa based on the photos (Fig 6). Accordingly, the presence/absence of this structure in all other species known in the genus should be confirmed to determine whether it can be used as a valid diagnostic character to separate species groups in the genus.

### Tribe Amblyseiini Muma, genus *Amblyseius* Berlese

#### 4. *Amblyseius aerialis* (Muma)

*Amblyseiopsis aerialis* [13]: 264.

*Amblyseius aerialis* (Muma); [33]: 91; [34]: 71; [35]: 15; [36]: 377; [37]: 238; [38]: 203; [39]: 3; [40]: 3; [41]: 609; [42]: 1136.

*Typhlodromus* (*Amblyseius*) *aerialis* [30]: 88.

Female (n = 5).

**Fig 7:**
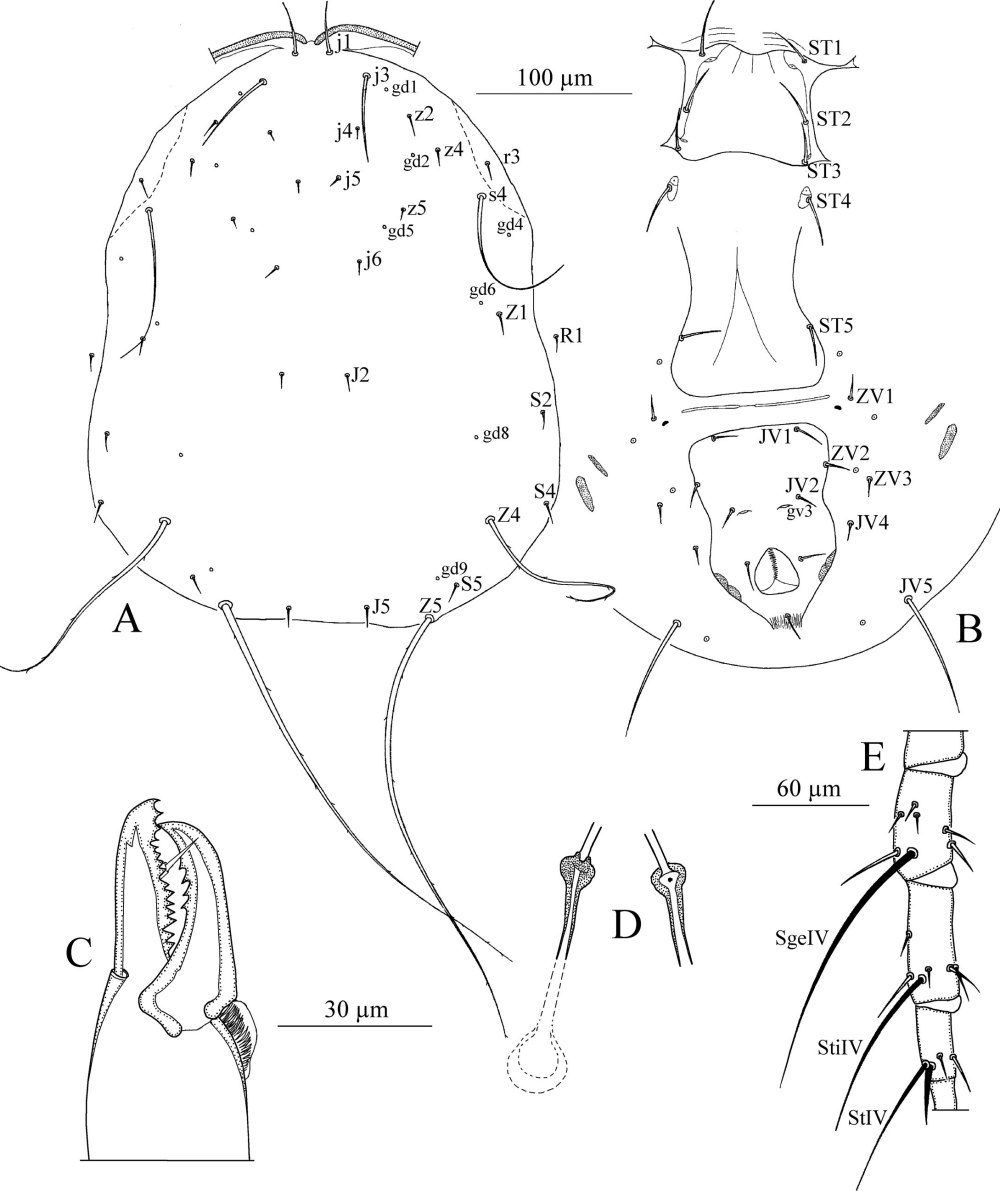
*Amblyseius aerialis* (Muma)–Female (A–E): A. Dorsal shield, B. Ventral idiosoma, C. Chelicera, D. Spermatheca, E. Leg IV. Scale bars = 100 μm for A, B; 30 μm for C, D; 60 μm for E (Photo credit: Ismail Döker).

*Dorsum* (Fig 7A). Dorsal setal pattern 10A:9B (*r3* and *R1* off shield in all specimen except, illustrated specimen where *r3* folded on the dorsal shield). Dorsal shield oval with a slight waist at level of *Z1*, smooth. Bearing seven pairs of rounded solenostomes (*gd1*, *gd2*, *gd4*, *gd5*, *gd6*, *gd8*, and *gd9*). Length of dorsal shield 419 (400–430), width (distance at level of *s4*) 271 (263–288), width (distance at level of *S2*) 321 (305–330). All dorsal setae smooth except *Z4* and *Z5* which have very little barbs. Measurements of dorsal setae as follows: *j1* 35 (33–37), *j3* 53 (49–55), *j4* 5, *j5* 5, *j6* 7 (6–8), *J2* 8 (8–9), *J5* 8 (8–9), *z2* 11 (10–12), *z4* 9 (8–9), *z5* 5 (5–6), *Z1* 9 (8–10), *Z4* 119 (110–125), *Z5* 291 (280–297), *s4* 105 (100–108), *S2* 10 (9–11), *S4* 12 (11–13), *S5* 13 (12–13), *r3* 13 (12–13), and *R1* 12 (11–13).

*Peritreme*. Long, extending beyond setae *j1*.

*Venter* (Fig 7B). Ventral setal pattern 14:JV–3:ZV. Sternal shield smooth except for a few anterior striations, lightly sclerotized with three pairs of setae (*ST1*, *ST2*, *ST3*), two pairs of poroids (*pst1* and *pst2*). Distance (*ST1*–*ST3*) 70 (68–71), width (*ST2*–*ST2*) 84 (78–87). Metasternal setae *ST4* and a pair of pores (*pst3*) on metasternal shields. Genital shield smooth; width at level of genital setae (*ST5*) 89 (83–93). Ventrianal shield smooth, bearing three pairs of pre-anal setae (*JV1*, *JV2*, and *ZV2*), a pair of para-anal (*Pa*) and a post-anal seta (*Pst*), and with a pair of crescentic solenostomes (*gv3*) posteromedian to *JV2*; distance *gv3*–*gv3* 26 (25–27). Length of ventrianal shield 127 (125–130), width at level of *ZV2* 90 (88–90), width at level of anus 87 (83–89) (widest level). Setae *JV4*, *JV5*, *ZV1*, *ZV3*, and four pairs of poroids on integument surrounding ventrianal shield. Two pairs of metapodal plates, primary 25 (23–25), and secondary 19 (18–21) in length. Setae *JV5* smooth, much longer than other ventral setae 83 (78–89) in length.

*Chelicera* (Fig 7C). Fixed digit 38 (36–40) long with 14 teeth, with *pilus dentilis*; movable digit 41 (40–42) long with four teeth.

*Spermatheca* (Fig 7D). Calyx tubular, 16 (15–17) in length; atrium bulbous larger than calyx and incorporated in the base of calyx, major duct long.

*Legs* (Fig 7E). Length of legs (base of coxae to base of claws) as follows: leg I 411 (375–425), leg II 337 (330–340), leg III 343 (335–350), leg IV 444 (435–450). Genua II, III, and IV each with seven setae. Macrosetae present in all legs. Measurements of macrosetae as follows: *SgeI* 47 (45–49), *SgeII* 43 (41–45), *SgeIII* 63 (60–68), *StiIII* 42 (40–44), *StIII* 34 (32–35), *SgeIV* 136 (133–138) *StiIV* 91 (86–94) and *StIV* 79 (75–81).

**Fig 8:**
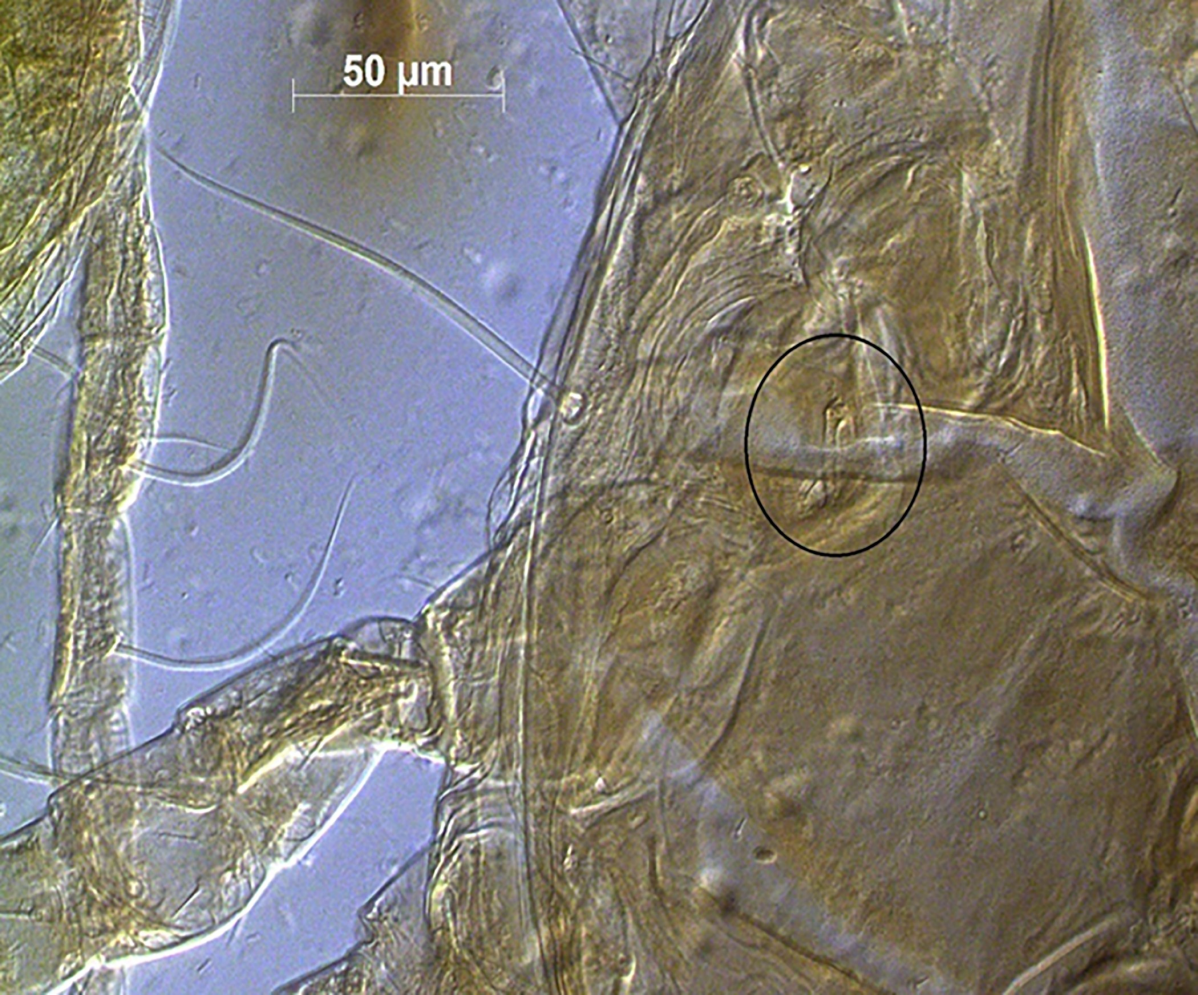
General view of spermatheca of *Amblyseius aerialis* (holotype) (Photo credit: Ronald Ochoa).

*Remarks*. *Amblyseius aerialis* description of Muma [13] was based on the material collected from leaves of *Citrus* sp. in Lucerne Park, Florida. Many morphological characters including setae measurements were not included in that original description. These measurements were provided for the specimens of different populations collected from many South American countries such as Argentina [39], Brazil [40], Colombia [34], Dominican Republic [41], Guadeloupe [36], Guyana [33], and Peru [42], but illustrations are absent in most papers except some partial drawings in De Leon [33] and Muma [13]. In addition, a full set of illustrations is available in Muma & Denmark [26] and Denmark & Muma [35]. However, the bulbous (enlarged) atrium of spermatheca is not clearly illustrated in the available literature [26, 33, 35, 43]. Therefore, we re-described this species based on the specimens collected from its type host *Citrus* sp. in Fort Pierce, Vero Beach, and Clermont, all close to its type location in Lucerne Park, Florida (170, 133, and 64 km, respectively) to provide evidence for additional diagnosis including a detailed illustration with drawings of the spermatheca and other characters. Some morphological characters and measurements of the specimens that we observed are very close to those provided by others [33, 35, 36, 40], nevertheless, there were several noticeable variations. For example, Muma [13] mentioned a smooth movable digit in the original description. However, our observation on the type materials confirmed four teeth on that cheliceral digit as mentioned in other redescriptions. The shape of the calyx of spermatheca also fits well with that of the holotype specimen (Fig 8).

#### 5. *Amblyseius curiosus* (Chant & Baker)

*Iphiseius curiosus* [44]: 11. *5*.

*Amblyseius curiosus* (Chant & Baker); [45]: 202; [26]: 64; [38]: 201.

*Amblyseius* (*Amblyseius*) *curiosus* (Chant & Baker); [35]: 35; [1]: 65.

Female (n = 5).

**Fig 9:**
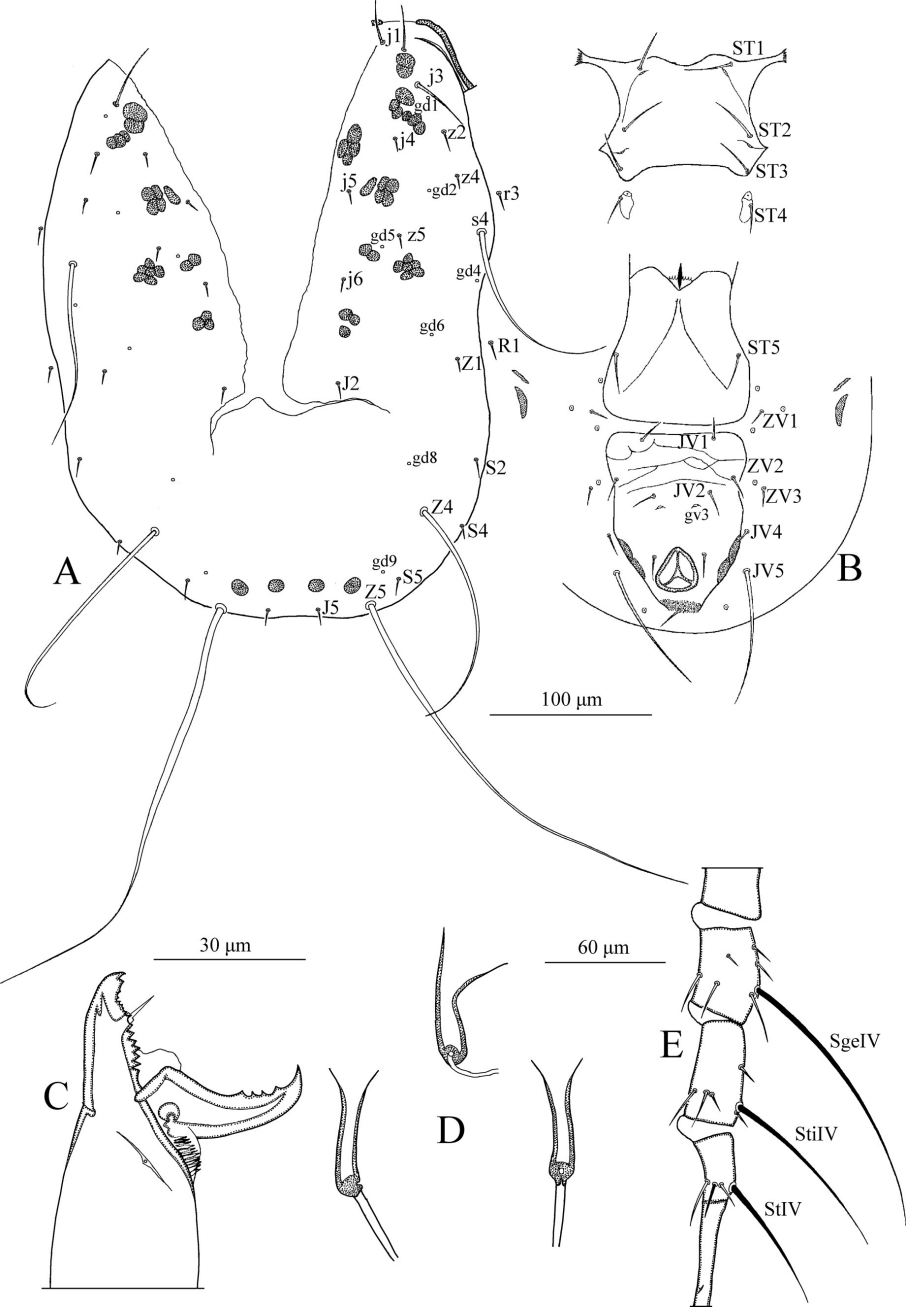
*Amblyseius curiosus* (Chant & Baker)–Female (A–E): A. Dorsal shield, B. Ventral idiosoma, C. Chelicera, D. Spermatheca, E. Leg IV. Scale bars = 100 μm for A, B; 30 μm for C, D; 60 μm for E (Photo credit: Ismail Döker).

*Dorsum* (Fig 9A). Dorsal setal pattern 10A:9B (*r3* and *R1* off shield). Dorsal shield oval with a slight waist at level of *Z1*, smooth. Bearing seven pairs of rounded solenostomes (*gd1*, *gd2*, *gd4*, *gd5*, *gd6*, *gd8* and *gd9*). Muscle-marks (sigilla) visible mostly on podosoma, length of dorsal shield 361 (320–388), width (distance at level of *s4*) 236 (220–250), width (distance at level of *S2*) 258 (250–265). All dorsal setae smooth. Measurements of dorsal setae as follows: *j1* 27 (26–29), *j3* 33 (32–34), *j4* 5 (4–5), *j5* 5 (4–5), *j6* 5 (4–5), *J2* 8 (7–8), *J5* 8 (7–8), *z2* 11 (10–13), *z4* 6 (5–8), *z5* 6 (5–8), *Z1* 6 (5–8), *Z4* 142 (140–145), *Z5* 304 (265–325), *s4* 131 (115–145), *S2* 6 (5–8), *S4* 6 (5–8), *S5* 11 (9–14), *r3* 10 (10–11), and *R1* 9 (8–10).

*Peritreme*. Long, extending to level of setae *j1*.

*Venter* (Fig 9B). Ventral setal pattern 14:JV–3:ZV. Sternal shield smooth, lightly sclerotized with three pairs of setae (*ST1*, *ST2*, *ST3*), two pairs of poroids (*pst1* and *pst2*). Distance (*ST1*–*ST3*) 65 (64–65), width (*ST2*–*ST2*) 79 (78–80). Metasternal setae *ST4* and a pair of pores (*pst3*) on metasternal shields. Genital shield smooth; width at level of genital setae (*ST5*) 79 (70–85). Ventrianal shield reticulated anteriorly, bearing three pairs of pre-anal setae (*JV1*, *JV2*, and *ZV2*), a pair of para-anal (*Pa*) and a post-anal seta (*Pst*), and with a pair of crescentic solenostomes (*gv3*) posterior to *JV2*; distance *gv3*–*gv3* 22 (20–23). Length of ventrianal shield 117 (103–127), width at level of *ZV2* 94 (88–98), width at level of anus 75 (73–78) (widest level). Setae *JV4*, *JV5*, *ZV1*, *ZV3*, and five pairs of poroids on integument surrounding ventrianal shield. Setae *JV5* smooth, much longer than other ventral setae 78 (65–83) in length.

*Chelicera* (Fig 9C). Fixed digit 29 (27–30) long with 15 teeth, with *pilus dentilis*; movable digit 32 (29–33) long with four teeth.

*Spermatheca* (Fig 9D). Calyx saccular flaring distally, 23 (22–23) in length; atrium incorporated in the base of calyx, a very small fork is visible in some specimens, major duct long and narrow.

*Legs* (Fig 9E). Length of legs (base of coxae to base of claws) as follows: leg I 393 (380–405), leg II 308 (303–315), leg III 306 (295–315), leg IV 388 (370–405). Genua II, III, and IV each with seven setae. Macrosetae present in all legs. Measurements of macrosetae as follows: *SgeI* 46 (45–48), *SgeII* 39 (35–42), *SgeIII* 65 (58–70), *StiIII* 43 (38–46), *StIII* 25 (24–26), *SgeIV* 151 (130–162) *StiIV* 96 (80–108) and *StIV* 71 (58–75).

**Fig 10:**
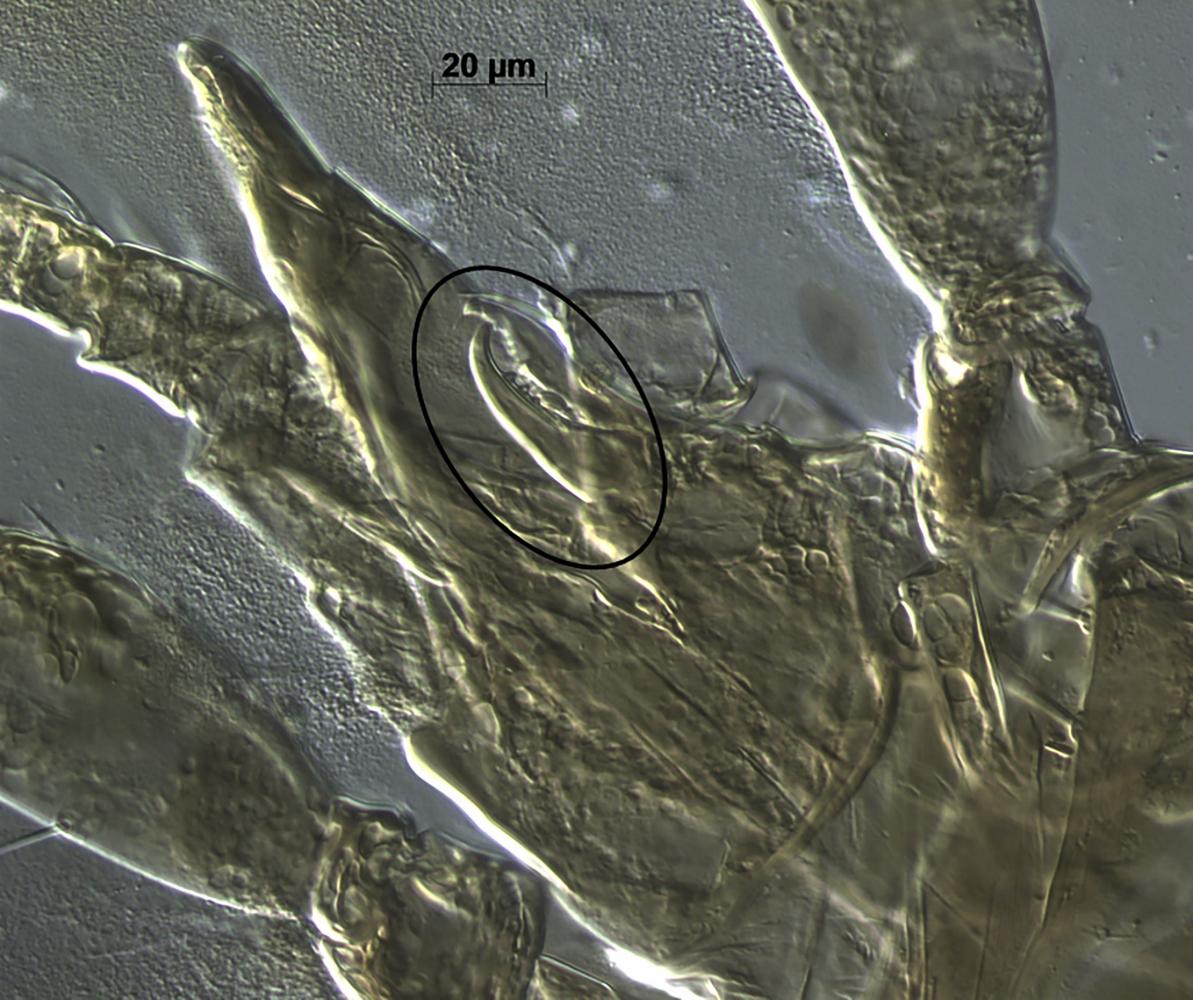
Chelicera of *Amblyseius curiosus* (holotype) (Photo credit: Ronald Ochoa).

*Remarks*. *Amblyseius curiosus* was described from Costa Rica based on the material collected from an unknown host shrub by Chant & Baker [44]. No measurements were provided in the original and the subsequent re-descriptions [26, 44] even though the original description presents clear drawings [44]. Denmark & Muma [35] provided a re-description based on a single specimen without measurements of some leg macrosetae. Therefore, we provided a complementary description. Morphological characters of the specimens examined in our study are in agreement with those of type materials. We noticed four teeth through examination of the photographs of the type specimens (Fig 10). Three are obvious in the photograph and the fourth next to the junction between the fixed and movable digit.

### Tribe Amblyseiini Muma, genus *Proprioseiopsis* Berlese

#### 6. *Proprioseiopsis carolinianus* (Muma, Metz & Farrier)

*Amblyseius carolinianus* [45]: 199.

*Proprioseiopsis carolinianus* (Muma, Metz & Farrier); [46]: 13; [1]: 197.

Female (n = 14).

**Fig 11:**
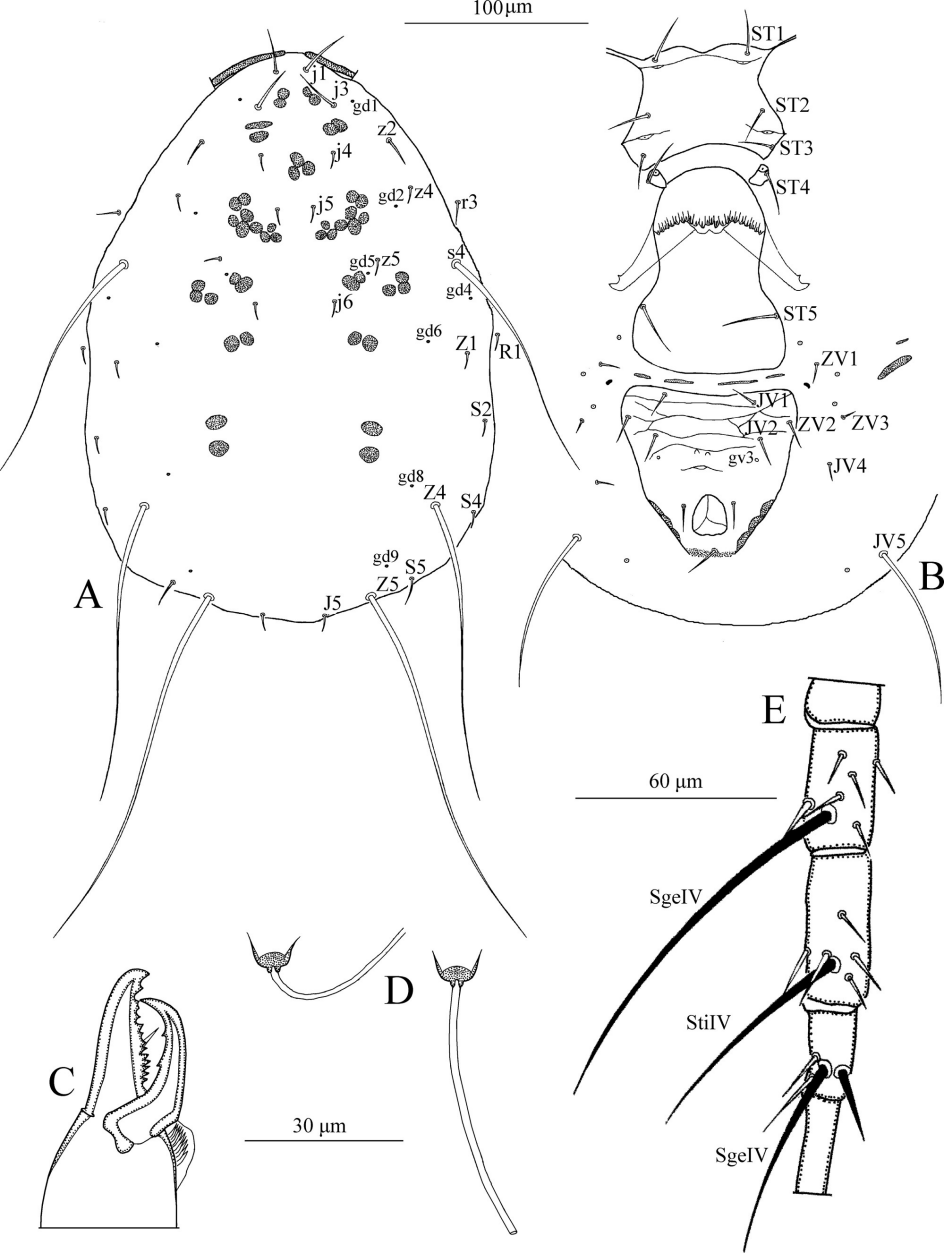
*Proprioseiopsis carolinianus* (Muma, Metz & Farrier)–Female (A–E): A. Dorsal shield, B. Ventral idiosoma, C. Chelicera, D. Spermatheca, E. Leg IV. Scale bars = 100 μm for A, B; 30 μm for C, D; 60 μm for E (Photo credit: Ismail Döker).

*Dorsum* (Fig 11A). Dorsal setal pattern 10A:8E (*r3* and *R1* off shield). Dorsal shield oval with a slight waist at level of *Z1*, smooth. Bearing seven pairs of rounded solenostomes (*gd1*, *gd2*, *gd4*, *gd5*, *gd6*, *gd8* and *gd9*). Muscle-marks (sigilla) visible on podosoma, length of dorsal shield 334 (300–360), width (distance at level of *s4*) 247 (195–268), width (distance at level of *S2*) 265 (230–280). All dorsal setae smooth. Measurements of dorsal setae as follows: *j1* 26 (20–30), *j3* 26 (23–28), *j4* 6 (5–8), *j5* 7 (5–8), *j6* 7 (5–8), *J5* 7 (5–8), *z2* 16 (10–18), *z4* 6 (5–8), *z5* 6 (5–8), *Z1* 6 (5–8), *Z4* 178 (165–187), *Z5* 254 (220–278), *s4* 149 (133–163), *S2* 6 (5–8), *S4* 6 (5–8), *S5* 13 (10–15), *r3* 9 (8–11), and *R1* 6 (5–8).

*Peritreme*. Long, extending to level setae *j1*.

*Venter* (Fig 11B). Ventral setal pattern 14:JV–3:ZV. Sternal shield smooth, lightly sclerotized with three pairs of setae (*ST1*, *ST2*, *ST3*), two pairs of poroids (*pst1* and *pst2*). Distance (*ST1*–*ST3*) 57 (53–59), width (*ST2*–*ST2*) 66 (63–69). Metasternal setae *ST4* and a pair of pores (*pst3*) on metasternal shields. Genital shield smooth; width at level of genital setae (*ST5*) 77 (70–82). Ventrianal shield reticulated, bearing three pairs of pre-anal setae (*JV1*, *JV2*, and *ZV2*), a pair of para-anal (*Pa*) and a post-anal seta (*Pst*), and with a pair of small rounded solenostomes (*gv3*) posterior to *JV2*; distance *gv3*–*gv3* 52 (48–55). Length of ventrianal shield 96 (85–101), width at level of *ZV2* 95 (83–100), width at level of anus 80 (68–85) (widest level). Setae *JV4*, *JV5*, *ZV1*, *ZV3*, and four pairs of poroids on integument surrounding ventrianal shield. Two pairs of metapodal plates, primary 22 (19–23), and secondary 8 (7–9) in length, Setae *JV5* smooth, much longer than other ventral setae 74 (65–84) in length.

*Chelicera* (Fig 11C). Fixed digit 28 (23–29) long with 13 teeth, with *pilus dentilis*; movable digit 29 (28–30) long with two teeth.

*Spermatheca* (Fig 11D). Calyx cup-shaped, slightly flaring distally, 5 (4–6) in length; atrium has a very small fork, major duct long.

*Legs* (Fig 11E). Length of legs (base of coxae to base of claws) as follows: leg I 345 (320–358), leg II 265 (245–280), leg III 266 (240–280), leg IV 343 (313–360). Genua II, III, and IV with eight, seven, and seven setae, respectively. Macrosetae present in all legs. Measurements of macrosetae as follows: *SgeI* 33 (30–36), *SgeII* 32 (27–35), *SgeIII* 47 (34–53), *StiIII* 28 (25–30), *StIII* 24 (20–26), *SgeIV* 119 (100–128) *StiIV* 70 (63–79) and *StIV* 60 (53–68).

**Fig 12:**
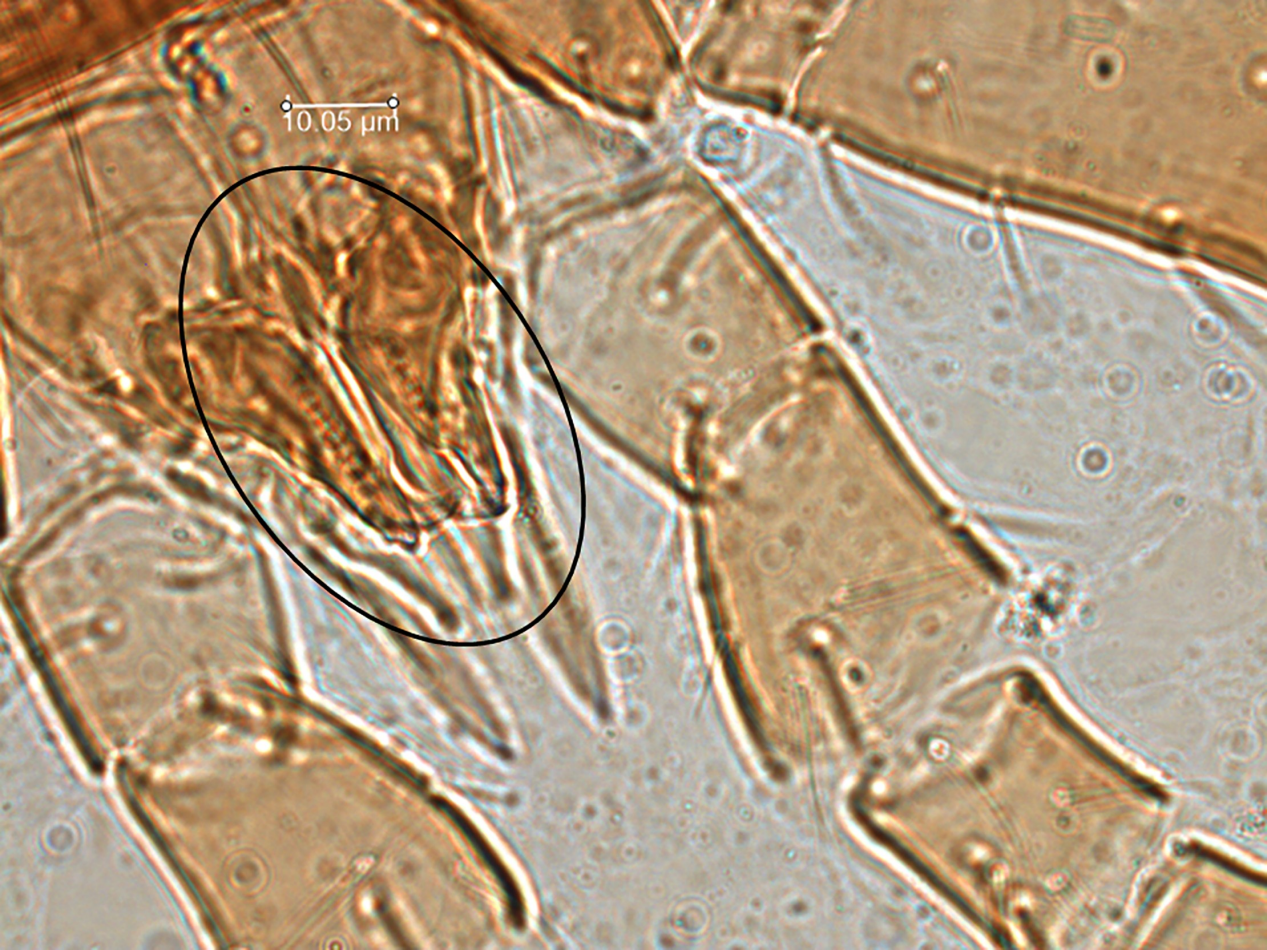
Chelicerae of *Proprioseiopsis carolinianus* (paratype) (Photo credit: Emilie Demard).

*Remarks*. *Proprioseiopsis carolinianus* was described based on two specimens collected from pine and oak litter from North Carolina by Muma *et al*. [45]. This original description is not well defined and there is no re-description available. Denmark & Evans [1] reported this species from Missouri, and they separated it from their new species, *P*. *paracarolinianus* Denmark & Evans based on some setae of single specimens of both species. Their measurements of *s4*, *Z4*, *Z5*, and *SgeIV* are 126, 151, 210, and 83 in *P*. *carolinianus* as opposed to 160, 176, 271, and 125 in *P*. *paracarolinianus*, respectively. Here, we provided a re-description based on 14 specimens all collected from the same habitat (leaf litter) and nearby the type location of *P*. *paracarolinianus*. A wide range of variation (the maximum values are 15–28% longer than the minimum values) in the aforementioned setae measurements is reported among the specimens examined in this study. When we compared our measurements with those provided by Denmark & Evans [1], our specimens were closer to *P*. *carolinianus* based on the minimum values and similar to *P*. *paracarolinianus* considering maximum values. Denmark & Evans [1] also mentioned that leg I is longer than leg IV in *P*. *carolinianus* while it is shorter than leg IV in *P*. *paracarolinianus*. In our specimens, measurements of these legs are subequal in length suggesting that setae measurements and relative length of leg I and leg IV provided by Denmark & Evans [1] may be invalid to separate *P*. *carolinianus* and *P*. *paracarolinianus*. Moreover, in the original description of *P*. *paracarolinianus*, three teeth on the movable digit of chelicerae were reported whereas all specimens that we examined have two teeth on that cheliceral digit. We were unable to confirm the presence of three teeth on the type material provided as chelicera were closed (Fig 12). However, a recent molecular study confirmed movable digit dentition as a diagnostic character in *Kampimodromus* [47], another genus in the subfamily Amblyseiinae. Based on our investigation and evidence we consider current specimens as *P*. *carolinianus*, which may be more common than *P*. *paracarolinianus*.

### Tribe *Euseiini* Chant & McMurtry, genus *Typhlodromalus* Muma

#### 7. *Typhlodromalus peregrinus* (Muma)

*Typhlodromus peregrinus* [13]: 270.

*Typhlodromus* (*Amblyseius*) *peregrinus* Muma; [30]: 97.

*Amblyseius peregrinus* (Muma); [48]: 60; [49]: 255; [34]: 73.

*Typhlodromalus peregrinus* (Muma); [50]: 221; [46]: 199; [39]: 15; [42]: 25; [51]: 42; [52]: 386.

Female (n = 5).

**Fig 13:**
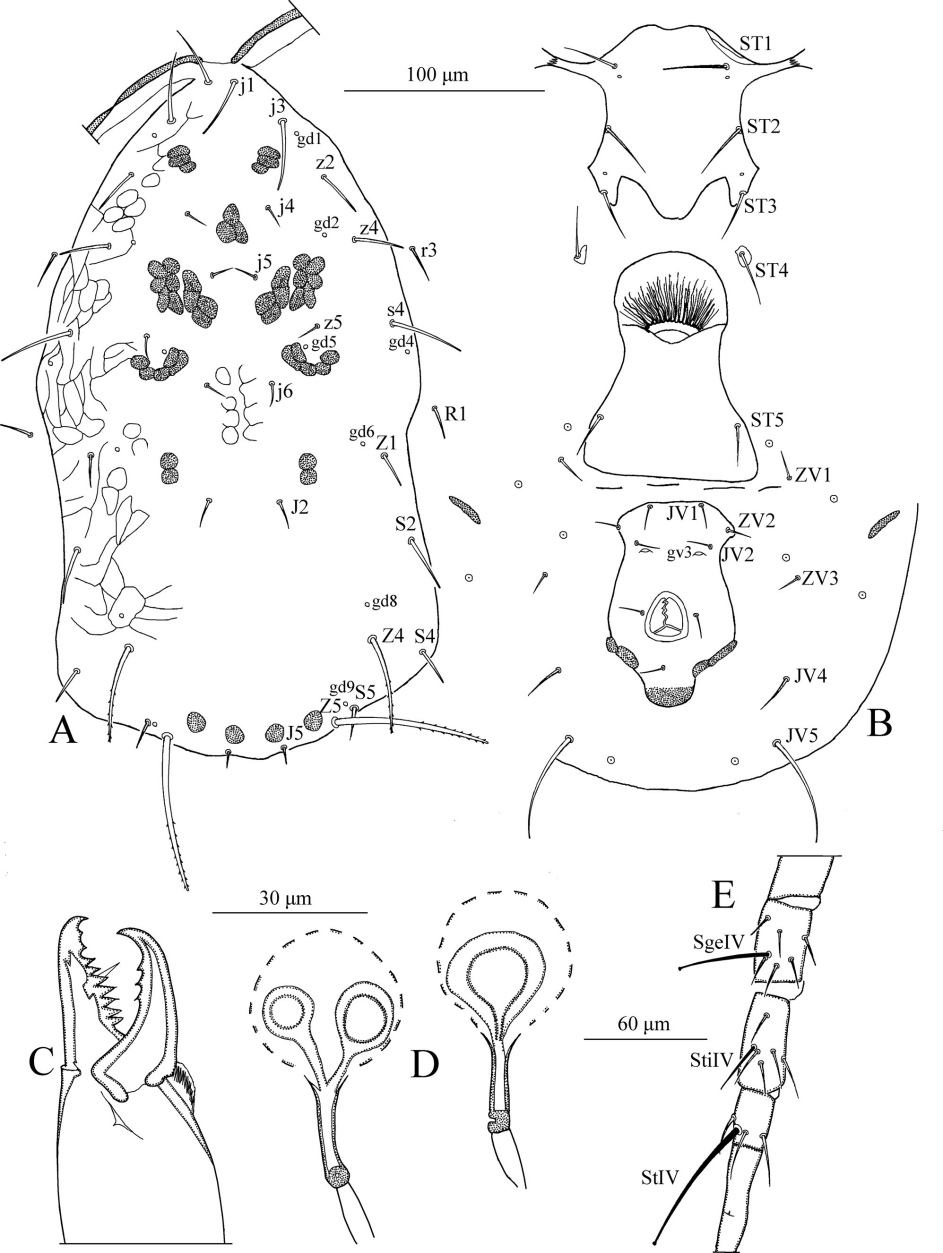
*Typhlodromalus peregrinus* (Muma)–Female (A–E): A. Dorsal shield, B. Ventral idiosoma, C. Chelicera, D. Spermatheca, E. Leg IV. Scale bars = 100 μm for A, B; 30 μm for C, D; 60 μm for E (Photo credit: Ismail Döker).

*Dorsum* (Fig 13A). Dorsal setal pattern 10A:9B (*r3* and *R1* off shield). Dorsal shield oval with a waist at level of *Z1*, rugose ornamentations are visible. Margin of dorsal shield indented at level of setae *S5* giving the posterior margin of the shield in a trilobate shape. Bearing seven pairs of rounded solenostomes (*gd1*, *gd2*, *gd4*, *gd5*, *gd6*, *gd8*, and *gd9*). Muscle-marks (sigilla) visible mostly on podosoma, length of dorsal shield 339 (325–347), width (distance at level of *s4*) 190 (170–222), width (distance at level of *S2*) 193 (182–210). Dorsal setae smooth except *Z4* and *Z5* which are strongly serrated. Dorsal setae somewhat stout. Measurements of dorsal setae as follows: *j1* 26 (24–28), *j3* 33 (30–35), *j4* 11 (10–13), *j5* 11 (10–12), *j6* 13 (12–14), *J2* 14 (13–16), *J5* 10 (9–11), *z2* 22 (20–24), *z4* 24 (21–26), *z5* 11 (10–12), *Z1* 16 (15–17), *Z4* 43 (40–45), *Z5* 73 (65–80), *s4* 37 (35–40), *S2* 28 (25–32), *S4* 21 (18–24), *S5* 13 (12–14), *r3* 19 (17–21), and *R1* 17 (15–18).

*Peritreme*. Long, extending to level of setae *j1*.

*Venter* (Fig 13B). Ventral setal pattern 14:JV–3:ZV. Sternal shield smooth, lightly sclerotized with three pairs of setae (*ST1*, *ST2*, *ST3*), two pairs of poroids (*pst1* and *pst2*), prominent posterior projection is present. Distance (*ST1*–*ST3*) 66 (64–69), width (*ST2*–*ST2*) 64 (60–68). Metasternal setae *ST4* and a pair of pores (*pst3*) on metasternal shields. Genital shield smooth; markedly wider than ventrianal shield; width at level of genital setae (*ST5*) 74 (72–80). Ventrianal shield vase-shaped; smooth, with a strong waist at level of *JV2*; Setae *JV2* and *ZV2* have migrated forwards to the prenal area; margins at level of anus covered with muscle marks; bearing three pairs of pre-anal setae (*JV1*, *JV2*, and *ZV2*), a pair of para-anal (*Pa*) and a post-anal seta (*Pst*), and with a pair of crescentic solenostomes (*gv3*) posteriomedian to *JV2*; distance *gv3*–*gv3* 27 (24–32). Length of ventrianal shield 111 (101–120), width at level of *ZV2* 62 (57–65), width at level of anus 67 (59–74) (widest level). Setae *JV4*, *JV5*, *ZV1*, *ZV3*, and five pairs of poroids on integument surrounding ventrianal shield. Setae *JV5* smooth, much longer than other ventral setae 50 (45–56) in length.

*Chelicera* (Fig 13C). Fixed digit 32 (28–34) long with nine teeth, with *pilus dentilis*; movable digit 32 (30–34) long with three teeth.

*Spermatheca* (Fig 13D). Calyx saccular flaring distally, 16 (15–18) in length; atrium nodular attached to the calyx without neck, major duct broad.

*Legs* (Fig 13E). Length of legs (base of coxae to base of claws) as follows: leg I 321 (315–330), leg II 275 (265–312), leg III 274 (255–300), leg IV 370 (345–390). Genua II, III, and IV each with seven setae. Macrosetae present in all legs. Measurements of macrosetae as follows: *SgeI* 18 (15–20), *SgeII* 20 (18–22), *SgeIII* 26 (24–31), *StiIII* 18 (16–19), *StIII* 20, *SgeIV* 40 (38–42) *StiIV* 26 (25–28) and *StIV* 69 (62–82). Setae *SgeII*, *SgeIII*, *SgeIV*, and *StIV* are knobbed apically.

*Remarks*. *Tyhlodromalus peregrinus* was described from Minneola, Florida, based on the material collected from orange leaves by Muma [13]. While setae measurements are absent in the original description, these measurements were provided for specimens of different populations collected from South American countries such as Argentina, Brazil, Colombia, Dominican Republic, Martinique, Guadeloupe, Guatemala, and Peru, as well as for type materials [52]. *Typhlodromalus aripo* De Leon [53] described from Trinidad and also reported from many South American countries shows a close affinity to *T*. *peregrinus*. Moraes & Mesa [34] pointed out that setae *z2* is 50% shorter than *z4* in *T*. *aripo* while it is only 20% shorter than *z4* in *T*. *peregrinus*. Furthermore, they also reported macrosetae *StIV* as setaceous and knobbed or blunt in *T*. *peregrinus* and *T*. *aripo*, respectively [34]. The *StIV* was also depicted as sharp-pointed in *T*. *peregrinus* by Chant & McMurtry [46]. Here, we provided for the first time a complementary description of *T*. *peregrinus* based on the materials collected from Clermont, a location very close (10 km) to its type location. As stated by Moraes & Mesa [34], we observed setae *z2* is 20% shorter than *z4* in most or both setae at subequal length. However, all specimens examined in this study have macrosetae *StIV* knobbed apically. Kreiter *et al*. [52] examined type materials of both *T*. *peregrinus* and *T*. *aripo* and suspected *T*. *aripo* as a junior synonym of *T*. *peregrinus*. The lengths of setae *z2* and *z4* are listed among the apomorphic characters for some genus (e.g. *Amblyseius*, *Transeius*) in the subfamily Amblyseiinae [38]. Hence, we believe that further molecular studies or crossbreeding experiments are essential to conclude whether these species are conspecific.

## Implications for biological control

We re-described seven species of predatory mites from the family Phytoseiidae, which lacked the use of important morphological features or information in the previous descriptions or re-descriptions.

There is a lack of knowledge regarding the food habits and predation efficiency of a large number of phytoseiid species. Little is known about the biology, ecology, behavior, food habits, and predation of the species redescribed here, except for *T*. *peregrinus* and *A*. *aerialis*. *Typhlodromalus peregrinus* is a prevalent species in citrus tree canopy and ground cover plants in Florida citrus orchards [54]. According to McMurtry *et al*. [55], it is a generalist predator with type III feeding habit. It has the ability to feed on a wide range of food sources including mites such as *Tetranychus urticae* Koch and *Panonychus citri* (McGregor) (Acari: Tetranychidae) and can be artificially reared using pollen of plant species such as *Malephora crocea* (Jaquin) (Family: Aizoaceae), *Quercus virginiana* Miller (Family: Fagaceae), and *Typha latifolia* (L.) (Family: Typhaceae) [56]. Peña [57] reported that *T*. *peregrinus* also feeds on the citrus rust mite, *Phyllocoptruta oleivora* (Ashmead) (Acari: Eriophyidae) but prefers the broad mite, *Polyphagotarsonemus latus* (Banks) (Acari: Tarsenomidae) in laboratory and glasshouse trials when both species are offered. *Typhlodromalus peregrinus* was reported as a dominant phytoseiid species in Alabama citrus orchards, but densities were too low to provide effective control of *P*. *citri* [58, 59].

*Amblyseius aerialis* is also a generalist predator with Type-III feeding habit [60]. It was first collected feeding on six-spotted spider mite, *Eotetranychus sexmaculatus*, and has since been found in citrus orchards from Guadeloupe, Dominican Republic, Florida, and Brazil [51, 54, 61, 62]. Studies showed that it can feed and reproduce on *T*. *urticae* [63, 64]. The oviposition rate of *A*. *aerialis* was shown to be high on *Raoiella indica* Hirst (Acari: Tenuipalpidae) and cattail pollen, *Typha dominguensis* [62, 64], as well as on African oil palm pollen (*Elaeis guineensis*), and coconut pollen *(Cocos nucifera)* (both Arecaceae) [62], and low on *Calacarus heveae* Feres (Acari: Eriophyidae) and *T*. *urticae* [64].

The other five species considered here were mainly collected from ground cover and leaf litter. *Amblyseius curiosus* and *P*. *carolinianus* could be classified as type III-e generalist predators [2], as they were mainly found in leaf litter. *Neoseiulus marinellus* and *N*. *planatus* are also generalist predators living in confined spaces on monocotyledonous plants [2], as most of the specimens were collected from mixed ground cover including grasses from the family Poaceae. There is no information on the feeding habits and other biological parameters (development time, reproduction, or survivorship) of these species. Therefore, research is needed to assess the potential of phytoseiids found in these specific microhabitats as biological control agents. *Neoseiulus baraki* and *N*. *paspalivorus* which are also generalist predators found in monocotyledonous plants have been found in association with the coconut mite, *Aceria guerreronis* (Eriophyidae) [2]. These species were able to feed and reproduce on their prey in laboratory conditions [65, 66]. Moreover, *N*. *cucumeris* a commercially available predatory mite extensively used in the past decade is a species originally found in the soil/litter habitats [2]. Therefore, it is likely that the provision of refugees and augmentative releases of *N*. *marinellus* and *N*. *planatus* may help to enhance their potential to target Eriophyidae species in citrus groves.
